# Prevalence and predictive value of sarcopenia in hospitalized patients with ischemic colitis

**DOI:** 10.1038/s41598-024-65243-6

**Published:** 2024-06-21

**Authors:** Byoung Chan Ahn, Min Sagong, Jeongseok Kim, Myeongsoon Park, Jinmok Bae, Jin Wook Lee, Yoo Jin Lee, Ju Yup Lee, Byung Kuk Jang, Woo Jin Chung, Kwang Bum Cho, Jae Seok Hwang

**Affiliations:** 1 Division of Gastroenterology, Department of Internal Medicine, Keimyung University School of Medicine, Dalgubeol-Daero, Dalseo-Gu, 1035 Daegu, Republic of Korea; 2 Zane Cohen Centre for Digestive Diseases, Joseph and Wolf Lebovic Health Complex, Mount Sinai Hospital, 60 Murray Street, Toronto, ON M5T 3L9 Canada

**Keywords:** Sarcopenia, Ischemic colitis, Colitis, Colon, Diseases, Gastroenterology, Health care

## Abstract

Ischemic colitis (IC) and sarcopenia are associated with aging and multiple comorbidities. We aimed to investigate the prevalence and predictive role of sarcopenia in patients with IC. We retrospectively analyzed 225 hospitalized patients (median age, 72 years; women, 67.1%; severe IC, 34.2%) who were diagnosed with IC between January 2007 and February 2022. Sarcopenia was defined as the skeletal muscle index at the third lumbar vertebra determined by computed tomography. It was present in 49.3% (n = 111) of the patients and was significantly associated with severe IC compared to those without sarcopenia (48.6% *vs*. 20.2%, *P* < 0.001). Sarcopenia was associated with extended hospitalization (median: 8 *vs*. 6 days, *P* < 0.001) and fasting periods (4 *vs*. 3 days, *P* = 0.004), as well as prolonged antibiotic use (9 *vs*. 7 days, *P* = 0.039). Sarcopenia was linked to a higher risk of surgery or mortality (9.0% *vs*. 0%, *P* = 0.001) and independently predicted this outcome (odds ratio [OR], 11.17; 95% confidence interval [CI], 1.24‒1467.65, *P* = 0.027). It was prevalent among hospitalized patients with IC, potentially indicating severe IC and a worse prognosis. This underscores the importance of meticulous monitoring, immediate medical intervention, and timely surgical consideration.

## Introduction

Ischemic colitis (IC) occurs owing to blood flow that is insufficient to maintain the metabolic function of colonocytes and it accounts for 75% of intestinal ischemia^1,2^. Risk factors for IC comprise advanced age, diabetes, dyslipidemia, cardiac arrhythmia, congestive heart failure, chronic kidney disease, chronic obstructive pulmonary disease, irritable bowel syndrome, peripheral vascular disease, constipation, and medications such as digitalis, aspirin, vasopressors, and oral contraceptives^3^. Additionally, a population-based cohort study in the United States found that the annual incidence of IC was 16.3 per 100,000 person-years, and it has increased over the past three decades^4^. The incidence of IC tends to increase with age, and is associated with various medical conditions, surgical history, and drug usage^1,5^. About 14%‒66% of patients with IC require surgery, and the mortality rate is 25%‒83% after emergency colectomy, with a significant risk of post-operative morbidities such as prolonged intubation, septic shock, and pneumonia^6–8^. Identifying poor prognostic factors for IC and determining early intervention would help to improve the outcomes of IC treatment^9^.

Sarcopenia is characterized by an age-related decline of skeletal muscle mass, quality, and strength that starts to arise around the age of 40 years^10,11^. The ranges of global prevalence of age-related and severe sarcopenia are 10%‒27% and 2%‒9%, respectively, depending on the definition^10^. Sarcopenia is not exclusive to older adults and is linked to various illnesses, post-operative status, and lack of physical activity^12,13^. Sarcopenia can result in health-related adverse outcomes such as falls, fractures, functional decline, physical disability, and even mortality, rendering it a major public health burden^14,15^. Moreover, sarcopenia predicts poor outcomes for patients with surgically-treated chronic illnesses and older patients^16,17^.

The incidence of multiple chronic comorbidities increases as the global population ages^18^ and might lead to an increase in IC and sarcopenia. However, an association between them has not yet been elucidated. Therefore, we aimed to define the prevalence and predictive value of sarcopenia in hospitalized patients with IC.

## Materials and methods

### Study population

We retrospectively reviewed hospitalized patients who had been diagnosed with IC at Keimyung University Dongsan Medical Center between January 2007 and February 2022. The inclusion criteria were: IC confirmed by colonoscopy or sigmoidoscopy and assessed by contrast-enhanced abdominopelvic computed tomography (CT) at presentation, or treated by surgery for complications of severe IC. The exclusion criteria comprised no inpatient treatment, IC diagnosed while in hospital for other illnesses, concomitant infectious colitis with the etiology proven by stool tests, previous or current colorectal cancer, history of colectomy, or missing clinical data or abdominopelvic CT images covering the third lumbar vertebra (L3) area. The Institutional Review Board (IRB) of Keimyung University Dongsan Hospital approved the study (Approval no: 2023–09-038) which proceeded according to the ethical principles enshrined in the Declaration of Helsinki (2013 amendment). The IRB of Keimyung University Dongsan Hospital waived the need for informed consent owing to the retrospective nature of the study.

### Data collection and CT protocol

The demographics of the patients, laboratory data, endoscopic and abdominopelvic CT findings, and histological results were extracted from the electronic medical records. The clinical data comprised body mass index (BMI), the American Society of Anesthesiologists (ASA) classification score, blood pressure, heart rate, body temperature, and presence or absence of abdominal rebound tenderness. Comorbidities were classified using the Charlson comorbidity index (CCI)^19^.

Abdominopelvic CT images were routinely acquired using 64- or 128-channel multidetector CT scanners (Siemens Medical Solutions Inc., Malvern, PA, USA; GE HealthCare Technologies Inc., Chicago, IL, USA) at 100‒120 kVp using automatic exposure control. Portal venous phase scans typically occurred at 70 s after an intravenous injection of an iodinated contrast agent. All images were reconstructed as 5-mm slice thickness without interslice gaps. A trained observer (BCA) reviewed the CT images and reports of IC to determine affected segments, bowel wall thickening or edema, pericolic infiltration, decreased contrast enhancement, and intramural pneumatosis^20^. Radiologists then reviewed discordant findings between the CT imaging data and reports to confirm involved lesions. Endoscopic findings of inflamed areas such as mucosal redness, erosion, and ulcer were also reviewed^21^. Ulcers were defined as “deep” when the depth the most inflamed area was > 3 mm. The left colon was defined as rectum, sigmoid colon, and the splenic flexure. The right colon was defined as the cecum, ascending colon, and hepatic flexure. The involvement of two segments was defined as IC found in any two of the left, transverse, and right colon. We also reviewed histopathologic findings, which are rarely diagnostic^9^, but IC was primarily diagnosed based on combined clinical, imaging, and endoscopic or surgical findings.

### Definition of sarcopenia

**Figure 1:**
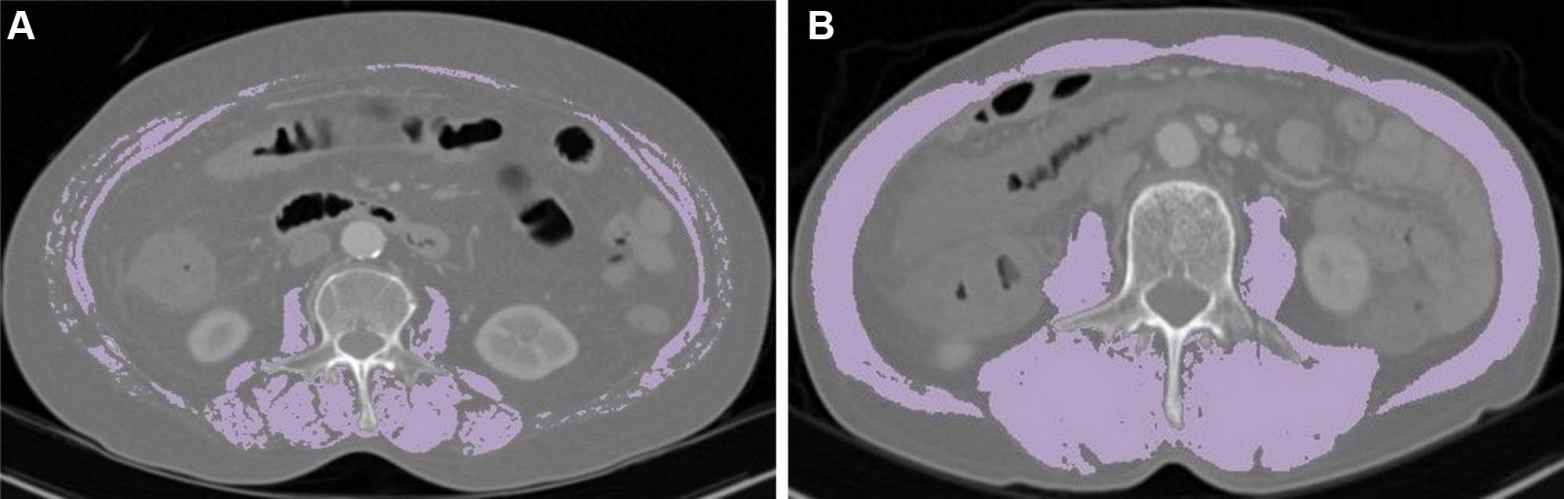
Computed tomography (CT) images of the skeletal muscle area (SMA, shown in bright purple) were obtained at the level of L3 vertebrae in sarcopenic (**A**) and non-sarcopenic (**B**) ischemic colitis patients using the "AsanJ-Morphometry" software^22^.

Skeletal muscle area (SMA), total fat area (TFA), visceral fat area (VFA), subcutaneous fat area (SFA), and bilateral psoas muscle areas were calculated from abdominopelvic CT images acquired at the L3 level using AsanJ-Morphometry™ software (Fig. 1)^22^. Skeletal muscle indices were defined as the total cross-sectional area of skeletal muscle at the L3 level divided by the BMI (SMA/BMI) or height squared (SMA/height^2^). The sex-specific cutoff values of SMA/BMI and SMA/height^2^ for defining sarcopenia were 4.97 and 3.46 cm^2^/m^2^ in men, and 39.8 and 28.4 cm^2^/m^2^ in women with reference to the results of a recent Asian population study^23^.

### Severity of IC

The severity of IC was evaluated according to the American College of Gastroenterology clinical guidelines^1^. Mild IC was defined as symptomatic segmental colitis not solely in the right colon without the risk factors for moderate IC listed below. Moderate IC was confirmed when up to any three of the following criteria were met: male sex, hypotension (systolic blood pressure < 90 mmHg), tachycardia (heart rate > 100 beats/min), white blood cell (WBC) counts < 15,000/µL, hemoglobin (Hb) < 12 g/dL, serum sodium < 136 mmol/L, blood urea nitrogen (BUN) > 28 mg/dL, lactate dehydrogenase (LDH) > 450 U/L, abdominal pain without rectal bleeding, or endoscopically proven colonic mucosal ulceration. Severe IC was defined as having > 3 of the criteria described above or any of the following: peritoneal signs, pneumatosis or portal venous gas on CT images, gangrene on endoscopic images, or pancolonic or isolated right colonic distribution on CT or endoscopic images. We assumed that LDH was within the normal range for 63 patients whose LDH values were not assessed.

### Statistical analysis

Categorical variables are expressed as numbers and percentages and were analyzed using chi-square or Fisher exact tests. Continuous variables are expressed as medians (interquartile range [IQR]) and were analyzed using Student t-tests or Wilcoxon rank sum tests depending on the distribution of variables. We identified predictors of mortality or the need for surgical intervention using Firth penalized-likelihood logistic regression to minimize analytical bias caused by the rarity of the event and complete separation^24^. Variables with *P* < 0.1 in the univariable analysis were further assessed using multivariable backward elimination. Values with two-sided *P* < 0.05 were considered statistically significant. All data were statistically analyzed using R version 4.2.3 (RStudio, Inc., Boston, MA, USA).

## Results

### Baseline characteristics

**Figure 2:**
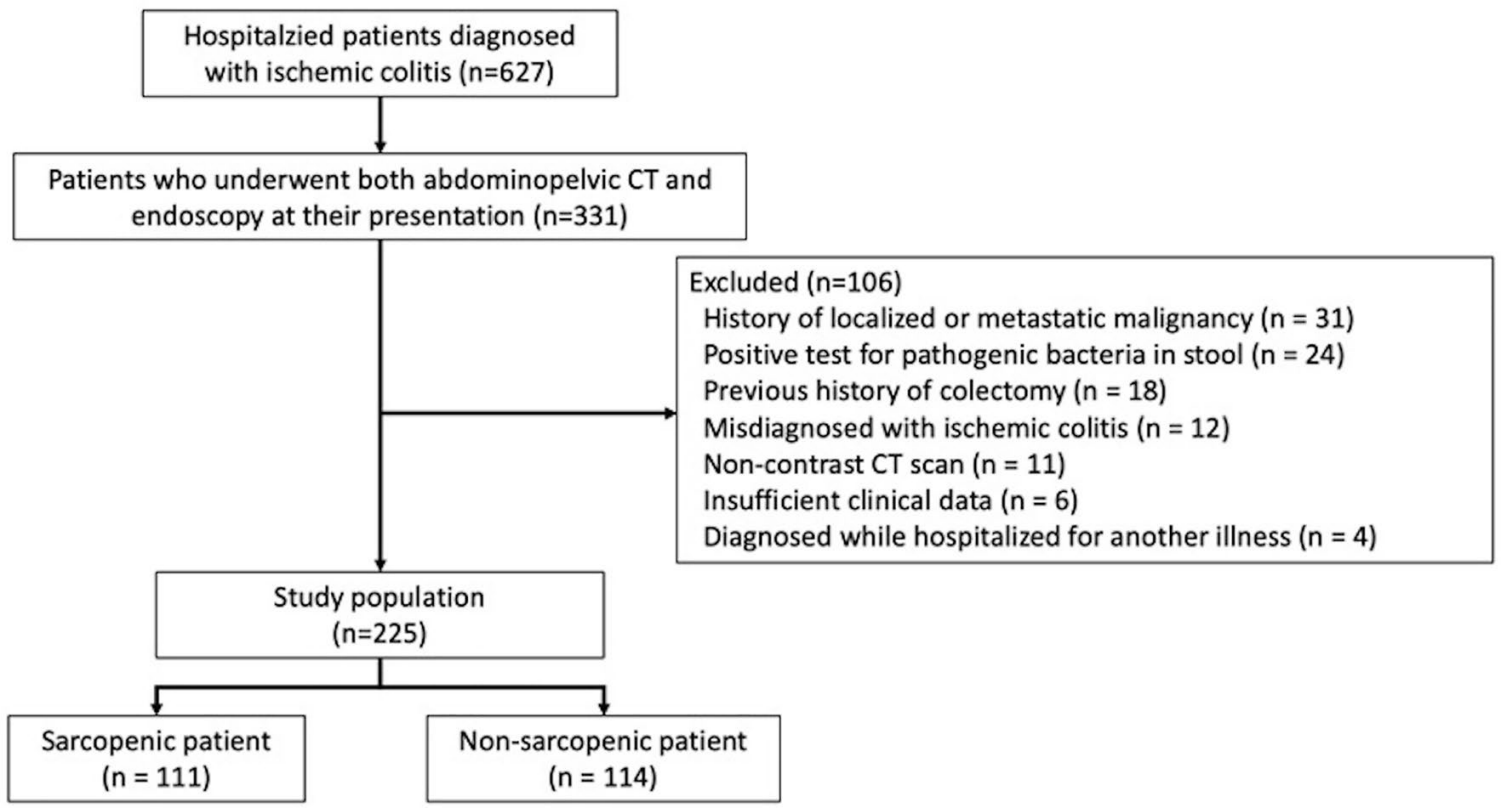
Flow chart of patient inclusion and exclusion. *CT* computed tomography.

Among the hospitalized patients who were diagnosed with IC (n = 627), 331 were assessed by abdominopelvic CT and endoscopy at the time of presentation, and 225 met the inclusion criteria (Fig. 2). The median (IQR) gap between when the patients presented and were assessed by endoscopy was 1 (0–2) days. The proportions of patients with mild, moderate, and severe IC were 5.8% (n = 13), 60.0% (n = 135), and 34.2% (n = 77), respectively. The median (IQR) age at IC diagnosis was older (75 [68‒79] *vs*. 70 [62‒77] years; *P* = 0.009), the proportion of males and the median CCI was higher (46.8% *vs*. 25.7%; *P* = 0.002 and 4 [3–5] *vs*. 3 [2–4]; *P* < 0.001, respectively) in patients with severe, than mild-to-moderate IC (n = 148). Tachycardia (13.0% *vs*. 2.7%; *P* = 0.006), hypotension (11.7% *vs*. 0%; *P* < 0.001), abdominal rebound tenderness (10.4% *vs*. 0%; *P* < 0.001), and deep colonic ulcers (49.4% *vs*. 8.8%; *P* < 0.001) were also more frequent in patients with severe IC. The neutrophil-to-lymphocyte ratio, BUN, creatinine, LDH, and CRP values were significantly higher, whereas lymphocyte counts, hemoglobin, albumin, and sodium values were lower. Among patients with severe IC, sarcopenia defined as SMA/BMI (70.1% *vs*. 38.5%; *P* < 0.001) or SMA/height^2^ (57.1% *vs*. 23.0%; *P* < 0.001) was more prevalent, and the results of the laboratory findings other than WBCs, were more unfavorable. Table 1 shows details of the patients’ characteristics.

**Table 1:** Clinical characteristics of the study population.

	Overall (n = 225)	Mild to Moderate IC (n = 148)	Severe IC (n = 77)	*P*
Age, year, median (IQR)	72 (64–78)	70 (62–77)	75 (68–79)	0.009
Sex, n (%)				0.002
Male	74 (32.9%)	38 (25.7%)	36 (46.8%)	
Female	151 (67.1%)	110 (74.3%)	41 (53.2%)	
BMI, kg/m^2^, median (IQR)	22.4 (20.7–24.8)	22.4 (20.8–24.8)	22.4 (20.0–24.0)	0.467
ASA grade, n (%)				0.069
1–2	134 (59.6%)	95 (64.2%)	39 (50.6%)	
3–4	91 (40.4%)	53 (35.8%)	38 (49.4%)	
History of abdominal surgery^a^, n (%)	41 (18.2%)	30 (20.3%)	11 (14.3%)	0.357
Comorbidities				
Diabetes, n (%)	50 (22.2%)	32 (21.6%)	18 (23.4%)	0.895
Hypertension, n (%)	124 (55.1%)	75 (50.7%)	49 (63.6%)	0.087
Ischemic heart disease, n (%)	37 (16.4%)	20 (13.5%)	17 (22.1%)	0.146
Cerebrovascular disease, n (%)	31 (13.8%)	15 (10.1%)	16 (20.8%)	0.046
Chronic kidney disease, n (%)	18 (8.0%)	4 (2.7%)	14 (18.2%)	< 0.001
Charlson comorbidity index, median (IQR)	3 (2–5)	3 (2–4)	4 (3–5)	< 0.001
Vital signs				
Fever, n (%)	33 (14.7%)	19 (12.8%)	14 (18.2%)	0.381
Tachycardia^b^, n (%)	14 (6.2%)	4 (2.7%)	10 (13.0%)	0.006
Hypotension^c^, n (%)	9 (4.0%)	0 (0%)	9 (11.7%)	< 0.001
Abdominal rebound tenderness, n (%)	8 (3.6%)	0 (0%)	8 (10.4%)	< 0.001
WBC count (10^3^/μL), median (IQR)	11.5 (8.7–14.4)	11.4 (8.8–13.9)	12.1 (7.8–16.1)	0.623
Lymphocyte count (10^3^/μL), median (IQR)	1.5 (1.0–2.0)	1.5 (1.2–2.0)	1.3 (0.6–1.9)	0.009
Neutrophil-to-lymphocyte ratio	6.2 (3.6–10.6)	5.7 (3.5–8.5)	7.3 (4.4–13.6)	0.015
Hemoglobin (g/dL), median (IQR)	12.9 (11.4–14.1)	13.2 (11.9–14.1)	11.7 (10.4–13.3)	< 0.001
BUN (mg/dL), median (IQR)	18 (14–26)	16 (13–22)	24 (16–35)	< 0.001
Creatinine (mg/dL), median (IQR)	0.8 (0.6–1.0)	0.7 (0.6–0.9)	1.0 (0.7–1.8)	< 0.001
LDH (U/L), median (IQR)	460 (387–570)	443 (381–512)	518 (417–641)	0.003
CRP (mg/dL), median (IQR)	1.9 (0.4–7.5)	1.5 (0.3–4.6)	5.1 (1.1–14.7)	< 0.001
Albumin (g/dL), median (IQR)	4.0 (3.5–4.3)	4.1 (3.7–4.3)	3.7 (3.1–4.1)	< 0.001
Alanine transaminase (IU/L), median (IQR)	16 (11–21)	16 (11–20)	14 (9–24)	0.645
Sodium (mEq/L), median (IQR)	138 (135–140)	138 (136–141)	137 (133–140)	0.004
Involved colonic segments assessed by CT scan				0.027
Left colon	118 (52.4%)	83 (56.1%)	35 (45.5%)	
Transverse colon	3 (1.3%)	1 (0.7%)	2 (2.6%)	
Right colon	4 (1.8%)	0 (0%)	4 (5.2%)	
Two segments	40 (17.8%)	27 (18.2%)	13 (16.9%)	
Whole colon	30 (13.3%)	16 (10.8%)	14 (18.2%)	
Not definite^d^	30 (13.3%)	21 (14.2%)	9 (11.7%)	
Involved colonic segments 1 assessed by combined results of CT, endoscopy, and surgical specimens				0.069
Left colon	134 (59.6%)	96 (64.9%)	38 (49.4%)	
Transverse colon	2 (0.9%)	0 (0%)	2 (2.6%)	
Right colon	5 (2.2%)	3 (2.0%)	2 (2.6%)	
Two segments	48 (21.3%)	29 (19.6%)	19 (24.7%)	
Whole colon	36 (16.0%)	20 (13.5%)	16 (20.8%)	
Involved colonic segments 2 assessed by combined results of CT, endoscopy, and surgical specimens, n (%)				0.207
Isolated right or whole colon	41 (18.2%)	23 (15.5%)	18 (23.4%)	
Others	184 (81.8%)	125 (84.5%)	59 (76.6%)	
Presence of colonic deep ulcer, n (%)	51 (22.7%)	13 (8.8%)	38 (49.4%)	< 0.001
Sarcopenia defined by SMA/BMI, n (%)	111 (49.3%)	57 (38.5%)	54 (70.1%)	< 0.001
Sarcopenia defined by SMA/height^2^, n (%)	78 (34.7%)	34 (23.0%)	44 (57.1%)	< 0.001
Duration of TPN use, days, median (IQR)	3 (2–4)	3 (2–4)	4 (2–7)	< 0.001

^a^Forty-one patients with a history of abdominal surgery (appendectomy, n = 16; gynecologic surgery, n = 12; cholecystectomy, n = 3; vascular surgery, n = 3; peritonitis surgery, n = 2; Cesarean section, n = 1, exploratory laparotomy, n = 1; liver segmentectomy, n = 1; cecectomy, n = 1).

^b^Tachycardia is defined as heart rate > 100 beats/min.

^c^Hypotension is defined as a systolic blood pressure < 90 mmHg. ^d^We identified 30 patients without definite IC involvement on CT images but were diagnosed by endoscopy. The distribution of IC was as follows: right colon, n = 4; transverse colon, n = 1; left colon, n = 22; involvement of 2 segments, n = 3.

*ASA* American Society of Anesthesiologists, *BMI* body mass index, *BUN* blood urea nitrogen, *CRP* C-reactive protein, *CT* computed tomography, *IC* ischemic colitis, *IQR* interquartile range, *LDH* lactate dehydrogenase, *SMA* skeletal muscle area, T*PN* total parenteral nutrition, *WBC* white blood cells.

### Body composition measures at the third lumbar (L3) vertebra

The values for SMI/BMI, and SMA/height^2^ were significantly lower in male and female patients with severe, than mild-to-moderate IC (Table 2). The prevalence of sarcopenia defined as SMI/BMI was higher in in males (72.2% [n = 26/36] *vs.* 39.5% [n = 15/38]; *P* = 0.009) and females (68.3% [n = 28/41] *vs.* 38.2% [n = 42/110]; *P* = 0.002) with severe IC. The trends were similar between males (63.9% [n = 23/37] *vs.* 34.2% [n = 13/38]; *P* = 0.020) and females (51.2% [n = 21/41] *vs.* 19.1% [n = 21/110]; *P* < 0.001), respectively, when sarcopenia was defined as SMA/height^2^.

**Table 2:** Comparison of body composition characteristics at L3 vertebra between patients with mild-to-moderate and severe ischemic colitis according to sex.

	Male			Female		
	Mild to Moderate IC (N = 38)	Severe IC (N = 36)	*P*	Mild to Moderate IC (N = 110)	Severe IC (N = 41)	*P*
Total fat area, cm^2^, median (IQR)	236.2 (109.3–389.0)	236.7 (154.6–313.8)	0.786	259.8 (199.0–336.6)	272.6 (192.5–335.6)	0.816
Subcutaneous fat area, cm^2^, median (IQR)	97.6 (64.5–135.9)	78.7 (59.0–118.7)	0.170	145.8 (106.6–183.2)	140.5 (105.4–175.2)	0.460
Visceral fat area, cm^2^, median (IQR)	102.5 (47.2–192.8)	127.4 (65.8–184.3)	0.501	100.4 (62.5–145.7)	100.5 (66.5–139.6)	0.647
SMA, cm^2^, median (IQR)	117.4 (100.9–136.0)	98.3 (82.3–116.7)	0.007	83.0 (68.6–91.7)	67.5 (59.5–76.9)	< 0.001
Right psoas muscle area, cm^2^, median (IQR)	66.6 (52.3–77.8)	48.1 (37.7–70.2)	0.022	35.2 (27.7–46.4)	33.5 (27.4–40.8)	0.118
Left psoas muscle area, cm^2^, median (IQR)	72.5 (58.0–86.7)	53.1 (41.0–68.6)	0.003	35.6 (29.5–48.6)	35.7 (25.4–44.6)	0.167
SMA/BMI, median (IQR)	5.3 (4.3- 5.9)	4.6 (3.9- 5.2)	0.011	3.6 (3.1- 4.1)	3.0 (2.5- 3.5)	< 0.001
Sarcopenia defined by SMA/BMI, n (%)	15 (39.5%)	26 (72.2%)	0.009	42 (38.2%)	28 (68.3%)	0.002
SMA/height^2^, cm^2^/m^2^, median (IQR)	43.2 (36.7–49.7)	35.4 (29.5–43.0)	0.003	34.0 (30.0–37.7)	28.1 (25.5–32.2)	< 0.001
Sarcopenia defined by SMA/height^2^, n (%)	13 (34.2%)	23 (63.9%)	0.020	21 (19.1%)	21 (51.2%)	< 0.001

*BMI* body mass index, *IC* ischemic colitis, *SMA* skeletal muscle area.

### Sarcopenia is associated with IC severity and prognosis

The prevalences of severe IC (48.6% *vs.* 20.2%; *P* < 0.001) and deep ulcers (31.5% *vs.* 14.0%; *P* = 0.003) were higher among patients with sarcopenia defined as SMA/BMI than in among those without sarcopenia (Table 3). Patients with sarcopenia also had significantly prolonged median hospital stays (8 [6–11] *vs.* 6 [5–8] days; *P* < 0.001), fasting duration (4 [3–7] *vs.* 3 [2–4] days; *P* = 0.004), and prolonged antibiotic use (9 [5–13] *vs.* 7 [4–12] days; *P* = 0.039). Nine (4.0%) patients underwent bowel surgery, and all had sarcopenia defined as SMA/BMI (Supplementary Table 1). Three of these nine patients died owing to IC-related sepsis. Among patients who were not surgically treated (n = 216), one (0.5%) died owing to aggravated pneumonia and colitis. This patient also had sarcopenia.

**Table 3:** Comparison of clinical characteristics and outcomes of ischemic colitis between sarcopenia and non-sarcopenia groups.

	Overall (n = 225)	Sarcopenia defined by SMA/BMI		Sarcopenia defined by SMA/height^2^			
		Sarcopenia (n = 111)	No sarcopenia (n = 114)	*P*	Sarcopenia (n = 78)	No sarcopenia (n = 147)	*P*
Severe IC, n (%)	77 (34.2%)	54 (48.6%)	23 (20.2%)	< 0.001	44 (56.4%)	33 (22.4%)	< 0.001
Presence of deep ulcer, n (%)	51 (22.7%)	35 (31.5%)	16 (14.0%)	0.003	27 (34.6%)	24 (16.3%)	0.003
Hospital stays, days, median (IQR)	7 (5–9)	8 (6–11)	6 (5–8)	< 0.001	8 (6–11)	7 (5–8)	< 0.001
Fasting duration, days, median (IQR)	4 (2–5)	4 (3–7)	3 (2–4)	0.004	4 (3–7)	4 (2–5)	0.016
Duration of antibiotic use, days, median (IQR)	8 (4–13)	9 (5–13)	7 (4–12)	0.039	9 (6–13]	7 (4–12)	0.028
Surgery, n (%)	9 (4.0%)	9 (8.1%)	0 (0%)	0.001	7 (9.0%)	2 (1.4%)	0.009
Death, n (%)	4 (1.8%)	4 (3.6%)	0 (0%)	0.058	3 (3.8%)	1 (0.7%)	0.121
Surgery or death, n (%)	10 (4.4%)	10 (9.0%)	0 (0%)	0.001	8 (10.3%)	2 (1.4%)	0.004

*BMI* body mass index, *IC* ischemic colitis, *SMA* skeletal muscle area.

### Predictors of mortality or surgical intervention

The findings of the multivariable logistic regression analysis showed that sarcopenia defined as SMA/BMI (OR, 11.17; 95% CI, 1.26‒1467.65; *P* = 0.027) was significantly associated with mortality or surgical intervention and the peritoneal irritation sign (OR, 35.10; 95% CI, 5.92‒290.36; *P* < 0.001) and BUN > 28 mg/dL (OR, 6.12; 95% CI, 1.35‒35.72; *P* = 0.019) (Table 4). Sarcopenia defined as SMA/height^2^ (OR, 8.33; 95% CI, 1.61‒77.13; *P* = 0.010) was also an independent predictor of mortality or surgery.

**Table 4:** Univariable and multivariable Firth’s penalized logistic regression analysis for predicting mortality or surgical intervention.

Variable	Univariable analysis		Multivariable analysis^a^		Multivariable analysis^b^	
	OR (95% CI)	*P*	OR (95% CI)	*P*	OR (95% CI)	*P*
Age (year)	1.04 (0.98–1.12)	0.167				
Female sex	1.72 (0.46–9.25)	0.442				
ASA 3–4 vs 1–2	1.01 (0.52–1.86)	0.979				
Charlson comorbidity index	1.14 (0.81–1.59)	0.430				
Fever	0.89 (0.09–4.07)	0.897				
Tachycardia	4.79 (0.84–19.87)	0.074				
Hypotension	8.18 (1.37–37.11)	0.024				
Peritoneal irritation sign	60.71 (13.02–332.41)	< 0.001	35.10 (5.92–290.36)	< 0.001	51.16 (8.88–400.13)	< 0.001
Isolated right or whole colon involvement vs others	1.31 (0.24–4.99)	0.718				
WBC > 15 × 10^3^/μL	1.02 (0.19–3.83)	0.983				
Hemoglobin < 12 g/dL	1.24 (0.33–4.21)	0.737				
BUN > 28 mg/dL	9.01 (2.56–38.44)	< 0.001	6.12 (1.35–35.72)	0.019	9.85 (2.12–65.76)	0.003
LDH > 450 U/L	1.14 (0.31–3.89)	0.833				
CRP > 0.5 mg/dL	2.06 (0.55–11.09)	0.298				
Sodium > 136 mmol/L	1.38 (0.33–4.83)	0.633				
Sarcopenia defined by SMA/BMI	23.69 (2.99–3060.71)	0.001	11.17 (1.26–1467.65)	0.027		
Sarcopenia defined by SMA/height^2^	7.01 (1.88–37.82)	0.003			8.33 (1.61–77.13)	0.010

^a^Multivariable analysis was conducted by incorporating SMA/BMI.

^b^Multivariable analysis was conducted by incorporating SMA/height^2^.

*ASA* The American Society of Anesthesiologists, *BMI* body mass index, *BUN* blood urea nitrogen, *CI* confidence interval, *CRP* C-reactive protein, *LDH* lactate dehydrogenase, *OR* odds ratio, *SMA* skeletal muscle area, *WBC* white blood cell.

## Discussion

We investigated associations between sarcopenia and IC, and the prognostic value of sarcopenia. The prevalence of sarcopenia in hospitalized patients with IC differed according to whether it was defined as SMA/BMI (49.3%) or SMA/height^2^ (34.7%). About 50% of the patients with sarcopenia had severe IC and 33% presented with deep ulcers. The prognosis of patients with sarcopenia was worse in terms of IC. Moreover, sarcopenia was identified as an independent predictor of mortality or surgical intervention. To our knowledge, this is the first study to determine an association between IC and sarcopenia.

Sarcopenia can be caused by infrequent skeletal muscle use, malnutrition, endocrine and metabolic changes, chronic inflammation, and consumptive disorder^25^. The prevalence of sarcopenia is relatively high among patients with stroke (42%), inflammatory bowel disease (41.6%), cancer (38.6%), liver cirrhosis (37.5%), heart failure (34%), rheumatoid arthritis (30.2%), end-stage renal disease (28.5%), chronic obstructive pulmonary disease (21.6%), chronic pancreatitis (17%‒62%), and type 2 diabetes (18%)^26^. We found here that sarcopenia is prevalent among hospitalized patients with IC, and that this tendency was more pronounced among those with severe IC.

Although the mechanisms underlying sarcopenia in IC require further investigation, we believe that shared risk factors associated with older age and comorbidities could explain the association between IC and sarcopenia^27,28^. Chronic inflammation, nutritional deficiency, lack of exercise, and dysbiotic gut microbiota in patients with inflammatory bowel disease^29^, might also contribute to the development of sarcopenia in IC. In particular, the intestinal microbiota is influenced not only by the aging process but also by several chronic diseases that interact reciprocally with intestinal ischemia according to preclinical animal studies^30,31^. Furthermore, the intestinal microbiota influences muscle function and quality through the gut-muscle axis in humans and experimental animals^26,32,33^. Appropriately-designed prospective studies are warranted to validate the relationship between IC and sarcopenia and identify the underlying mechanisms.

Sarcopenia can lead to impaired physical function, a diminished quality of life, increased risk of complications, and increased short- and long-term mortality^27,34,35^. The present findings also indicated that sarcopenia can predict a poor prognosis in patients with IC. This finding was in line with the established predictors, peritoneal signs, LDH > 450 U/L, bilateral or right-sided distribution, hepatitis C positivity, male sex, and chronic kidney disease^9^. This implies that patients with sarcopenia and IC are at a higher risk of treatment failure with conservative management, underscoring the importance of careful monitoring and proactive treatment approaches.

This study has some limitations. Firstly, we defined sarcopenia based on L3 CT images because most hospitalized patients with IC at our institution had been evaluated by CT. This method is valuable because muscle can be readily quantified from other body components. However, it’s worth noting the absence of a gold standard for diagnosing sarcopenia^36^. We acknowledge that the lack of measurement of skeletal muscle using dual energy X-ray absorptiometry, magnetic resonance imaging, and electrical bioimpedance, or functional indices, such as physical strength and frailty, might hinder the interpretation and generalization of our findings. Secondly, cutoff values for sarcopenia vary across studies and populations^37,38^. We addressed this issue by referring to a recently published study of a large Asian population in which the cutoff point for sarcopenia was defined as a T-score < 2.0^23^. In that study, SMA/BMI was proposed as the most reliable CT index for reflecting age-related muscle changes and achieving a high diagnostic yield^23^. Thirdly, our study population did not include patients with IC who were not admitted to hospital, which might limit the generalizability of our findings. However, IC is usually a milder disease that responds well to conservative management when hospitalization is not required. Therefore, the clinical implications of identifying sarcopenia in this group might not be as significant. Finally, since we included patients with IC who underwent endoscopy and CT, we might have excluded some patients with severe IC who did not undergo endoscopy owing to unstable vital signs, peritoneal signs, and ominous radiologic findings. However, to mitigate the risk of excluding those patients, we also included patients with severe IC that was surgically treated without endoscopic evaluation and whose diagnoses were further confirmed by clinical, imaging, and surgical pathology findings.

In conclusion, we found that the prevalence of sarcopenia is higher in hospitalized patients with, than without IC. We also uncovered a significant association between sarcopenia and IC severity, with sarcopenia being an independent predictor of mortality or surgical intervention. These findings suggest the need for meticulous monitoring, prompt medical treatment, and timely surgical consultation, particularly for patients with IC and sarcopenia.

### Supplementary Information

Supplementary Information.

## Data Availability

The datasets analyzed during the current study are available from the corresponding author upon reasonable request.
