# Supplementary material for: Prevalence and predictive value of sarcopenia in hospitalized patients with ischemic colitis
Source: Sci Rep. 2024 Jun 21;14:14352. doi: 10.1038/s41598-024-65243-6 (PMC11192930; doi:10.1038/s41598-024-65243-6)
Supplement: Supplementary file 1 — Supplementary Information. [file 41598_2024_65243_MOESM1_ESM.docx]

Supplementary Table 1. Clinical characteristics and outcomes of the patients with ischemic colitis who underwent surgery

| **No** | **Age** | **Sex** | **Previous illness or condition** | **BMI** | **Presence of sarcopenia defined by SMA/BMI** | **Involved segments** | **Bowel perforation** | **Operation** | **Deceased or alive** | **Cause of death** |
| --- | --- | --- | --- | --- | --- | --- | --- | --- | --- | --- |
| 1 | 78 | F | Hypertension, dyslipidemia, osteoporosis, and history of cholecystectomy due to cholecystitis | 21.1 | Yes | Lt & Rt colon | No | Subtotal small bowel resection, right hemicolectomy, and double barrel jejuno-colostomy on HD 1 | Death on HD 3 | IC-related |
| 2 | 76 | F | Osteoarthritis | 24.0 | Yes | Transverse colon & Lt colon | Sigmoid colon | Hartmann’s operation on HD 1 | Death on HD 2 | IC-related |
| 3 | 76 | F | Hypertension and diabetes | 20.0 | Yes | Whole colon | Transverse colon | Segmental resection of transverse colon and transverse colostomy on HD 1 | Death on HD 9 | IC-related |
| 4 | 76 | F | Hypertension | 22.7 | Yes | Lt colon | Sigmoid colon | Hartmann’s operation on HD 1 | Alive, discharged on HD 35 | NA |
| 5 | 69 | M | Hypertension and end-stage renal disease | 18.4 | Yes | Lt colon | Sigmoid colon | Hartmann’s operation on HD 1 | Alive, discharged on HD 22 | NA |
| 6 | 73 | F | Hypertension, diabetes, congestive heart failure, end-stage renal disease, and history of cholecystectomy due to cholecystitis | 24.0 | Yes | Whole colon | No | Ileocecectomy and ileostomy on HD 8 | Alive, discharged on HD 27 | NA |
| 7 | 80 | F | Hypertension and diabetes | 22.2 | Yes | Transverse colon & Lt colon | No | Lt colectomy and transverse colostomy on HD 3 | Alive, discharged on HD 16 | NA |
| 8 | 80 | F | Acute coronary syndrome | 25.4 | Yes | Transverse colon & Lt colon | Splenic flexure | Lt hemicolectomy on HD 8 | Alive, discharged on HD 15 | NA |
| 9 | 72 | F | Acute coronary syndrome and osteoporosis | 23.4 | Yes | Transverse colon & Lt colon | Sigmoid colon | Hartmann`s operation on HD 1 | Alive, discharged on HD 22 | NA |

BMI, body mass index; F, female; HD, hospital day; Lt, left; M, male; NA, not applicable; Rt, right; SMA, skeletal muscle area
